# Expression Analysis of the *TdDRF1* Gene in Field-Grown Durum Wheat under Full and Reduced Irrigation

**DOI:** 10.3390/genes13030555

**Published:** 2022-03-21

**Authors:** Arianna Latini, Cristina Cantale, Karthikeyan Thiyagarajan, Karim Ammar, Patrizia Galeffi

**Affiliations:** 1 Italian National Agency for New Technologies, Energy and Sustainable Economic Development, ENEA, Casaccia Research Center, 00123 Rome, Italy; arianna.latini@enea.it (A.L.); cantalec@gmail.com (C.C.); pltbiotechtkarthi2018@gmail.com (K.T.); 2 International Maize and Wheat Improvement Centre(CIMMYT), Texcoco 56237, Mexico; k.ammar@cgiar.org

**Keywords:** durum wheat, expression profiles, field trials, qRT-PCR, *TdDRF1* gene, transcription factors, *Wdhn13*

## Abstract

Some of the key genes and regulatory mechanisms controlling drought response in durum wheat have been identified. One of the major challenges for breeders is how to use this knowledge for the achievement of drought stress tolerance. In the present study, we report the expression profiles of the *TdDRF1* gene, at consecutive plant growth stages, from different durum wheat genotypes evaluated in two different field environments. The expression of a possible target gene (*Wdnh13*) of the *TdDRF1* gene was also investigated and analogies with the transcript profiles were found. The results of the qRT-PCR highlighted differences in molecular patterns, thus suggesting a genotype dependency of the *TdDRF1* gene expression in response to the stress induced. Furthermore, a statistical association between the expression of *TdDRF1* transcripts and agronomic traits was also performed and significant differences were found among genotypes, suggesting a relationship. One of the genotypes was found to combine molecular and agronomic characteristics.

## 1. Introduction

Unmitigated climate change due to increasing greenhouse gas emissions will have an adverse impact on plant growth and crop yield in some areas of the world, including through the more frequent occurrences of drought stress [1,2]. To mitigate against water scarcity and/or irregular availability and to enhance the sustainability of global food production, it is necessary to explore avenues for producing more food with proportionally less water [3]. Cereals are our dominant source of food, with wheat playing a major contribution to human diet and health [4]. Durum wheat (*Triticum turgidum* var. *durum*) is largely cultivated in the Mediterranean basin and other semi-arid and marginal areas, with the milled product being used mainly for making pasta and other staple foods.

Plants deploy complex mechanisms to cope with stresses like dehydration. Several genes have been described that are activated at the transcriptional level, with *cis*- and *trans*-acting factors involved in the expression of dehydration responsive genes [5]. The dehydration responsive element binding (DREB) family of transcription factors (TFs) represents one of the major players involved in abiotic (dehydration, cold, high salinity) stress responses [6]. The DREB proteins interact with the *drought-responsive element* (DRE) motif in the promoter regions of many stress-inducible genes and belong to the larger AP2/ERF (APETALA2/ethylene-responsive factor) family, as the DNA binding and recognition is mediated by the Apetala2 (AP2) domain [6,7]. TFs are therefore good candidates for improving crop tolerance to drought because of their role as master regulators of several clusters of genes [8,9,10].

A *DREB2*-related gene, namely *TdDRF1* (*Triticum durum dehydration responsive factor 1*), was isolated in durum wheat and reported as producing three forms of transcript through alternative splicing (AS): *TdDRF1.1* and *TdDRF1.3*, encoding putative TFs containing the AP2/EREBP DNA-binding domain and the nuclear localization signal (NLS), and *TdDRF1.2*, encoding a putative abortive protein lacking both the AP2 domain and the NLS. *TdDRF1* gene expression was linked to the plant response to water deficit [11] and its analysis in different genotypes of durum wheat and one triticale cultivar under greenhouse conditions and subjecting plants to a moderate dehydration stress resulted in different genotypic behaviours [12]. Furthermore, a preliminary study of the field expression of *TdDRF1* was also carried out, analysing durum wheat and triticale lines in a short time-course with five sampling points [13]. The above-mentioned studies revealed that *TdDRF1* gene expression had an important genotype-dependence, as also found for other transcription factor genes controlling plant response to abiotic stress [14,15] or key metabolic pathways [16,17]. The relationships among the expression patterns of the three transcripts and the phenotype response to water stress in a complex field environment are still largely unknown, highlighting the need for further research to gain more insight into the gene expression under realistic environmental conditions.

The aim of the present work was to investigate the molecular behaviour of the *TdDRF1* gene in the field during a time-course drought stress experiment in six durum wheat genotypes, and to find whether there was a relationship between the expression of the *TdDRF1* gene and one possible downstream target (*Wdhn13* gene). The association between transcript profile analysis and agronomic performance was also explored.

## 2. Materials and Methods

### 2.1. Plant Materials

Six durum wheat genotypes were used in the field experiments (Table 1).

Duilio, Creso, and Colosseo are Italian commercial varieties. The other three genotypes were developed by the CIMMYT program in Mexico, with Barnacla and AEL being advanced experimental lines and Altar C84 being a high yielding variety commercially released in Mexico and other countries.

### 2.2. Field Experiments

The field trial was conducted at the CIMMYT experimental station (Campo Experimental Norman Ernest Borlaug, CENEB) near Cd. Obregón (Sonora, Mexico) during the cropping season of 2010. The experiment was arranged in a randomized complete block design with 4 replicates and plots of 3.36 m^2^ for each of the two irrigation treatments or testing environments. The two different irrigation conditions were full irrigation (FI), with 550–600 mm of total water supplied by gravity irrigation during the full crop cycle, and reduced irrigation (RI), with 220–250 mm of total water applied through a drip system, all before heading. In both irrigation conditions plots were fertilized optimally as per the site-specific agronomic recommendations using a total of 250 units of nitrogen in the form of urea (50 units at sowing, 100 units at first node, and 100 units at the end of tillering) and phosphorus (50 units applied at sowing). Plots were maintained free of diseases and pests via the uniform application of fungicide and insecticide.

### 2.3. Agronomic Traits

After mechanical harvest of the whole plots, grain yield and thousand kernel weight were determined and considered in relation with the *TdDRF1* expression profiles.

### 2.4. RNA Extraction

A time-course experiment was designed with a sampling schedule consisting of 7 collection stages (T1 to T7), as reported in Table 2.

This schedule was planned to include the whole growing period, from heading to harvest. For each sampling, ten representative flag leaves were harvested for each plot, pooled together, immediately frozen in liquid nitrogen, and subsequently stored at −80 °C prior to RNA extraction. Total RNA was extracted from the leaves using the TRIzol^®^ Reagent (Invitrogen, Carlsbad, CA, USA) in accordance with the manufacturer’s instructions, and lyophilized. Lyophilized RNA samples (each yielding being approximately 25–35 µg of total RNA) were then resuspended in nuclease-free sterile water, qualitatively assessed by agarose gel electrophoresis, and quantified with a NanoDrop ND-1000 Spectrophotometer (NanoDrop Technologies, Wilmington, NC, USA).

### 2.5. Reverse Transcription, Pre-Amplification, and qRT-PCR

A set of specific primers, designed using the Assay-By-Design software (Applied Biosystems) with a view to obtain three specific and distinguishable fragments corresponding to each *TdDRF1* transcript, were used [11] (Supplementary Figure S1).

Supplementary Figure S2 is a schematic representation of the complete procedure of reverse transcription, pre-amplification, and qRT-PCR. The pre-amplification step was included to optimize the *real-time* reactions. A High-Capacity cDNA Reverse Transcription Kit (Applied Biosystems, Foster City, CA, USA) was used for the reverse transcription reactions. Samples (20 µL) contained 2 µL of 10× RT Buffer, 0.8 µL of 25× dNTP Mix (100 mM), 2 µL of 10× RT Random Primers, 1 µL of RNase Inhibitor (20 U/µL), and 2 µg of total RNA in nuclease-free water. The thermal cycling conditions were 10 min at 25 °C, 2 h at 37 °C, and 5 min at 85 °C and at 4 °C. Pre-amplification reactions (50 µL) contained 2 µL of TaqMan^®^ PreAmp Master Mix 2× (Applied Biosystems), 12.5 µL of pooled assay mix (0.2×, each assay), and 250 ng of cDNA sample in nuclease-free water. Reactions were held at 95 °C for 10 min and then at 95 °C for 15 s and 60 °C for 4 min 14 times. The resulting pre-amplified reactions were then diluted (1:20) in 1× TE buffer and used as the starting material for the subsequent Custom TaqMan^®^ Gene Expression Assays (Applied Biosystems) for the three target transcripts and the endogenous control carried out in the Applied Biosystems 7300 Real-Time PCR System. The final volume (20 µL) of a single PCR reaction contained 10 µL of 2× TaqMan^®^ Universal PCR Master Mix with AmpErase^®^ UNG (Applied Biosystems), 1 µL of 20× Custom TaqMan^®^ Gene Expression Assay (Applied Biosystems), and 2 µL of diluted pre-amplified product as a template. Samples were run in three biological replicates (from three randomized plots) and three technical replicates.

For endogenous control of relative quantification, different wheat genes were tested: *18S rRNA*, *TaSNK1* [18], *actin*, *Ta2291*, and *Ta2776* [19]. The GeNorm algorithm was used to calculate the gene-stability value (*M*) for all reference genes according to:

(1) $$Mj=\frac{\sum_{k=1}^{n}Vjk}{n-1}$$

where *Vjk* represents the arithmetic mean of all pairwise variations [19]. As the gene with the lowest *M* values showed the most stable expression, *TaSNK1* was used in our qRT-PCR assays (Supplementary Table S1).

A relative quantification of the *TdDRF1* transcripts was obtained using the ΔΔC_T_ method [20] for each sample and results were expressed as normalized relative quantity (NRQ). Furthermore, each expression profile was also calculated as the log2 value of the fold change (FC) (abundance under stress/abundance under control) for each transcript and time.

### 2.6. Transcripts of the Wdhn13 Gene

Based on the literature, the *Wdhn13* gene was chosen as a putative target of the regulation by the *Td*DRF1 transcription factor [21,22], using the sequence from *Triticum aestivum*, locus AB297677, deposited in NCBI in 2007. A qRT-PCR analysis was performed on RNA samples of AEL and of Barnacla collected from both the FI and RI conditions during the time-course. The following pair of primers was used: FOR 5′-GATGGCAACTACGGGAAGTC-3′ and REV 5′-GCAGCTTGTCCTTGATCTTG-3′, amplifying an 88 bp cDNA fragment, which was cloned for the setup of the standard curve. qRT-PCR reactions were performed using SYBR green technology in accordance with the procedure reported by Vítámvás and colleagues [23].

### 2.7. Statistical Analyses

All statistical analyses were carried out with IBM SSPS Statistics 23. The analysis of variance (ANOVA) for each parameter was performed at a 95% confidence level and the significant difference between means was tested using Tukey’s method when applicable. Furthermore, the significance of contrast between the up-regulated and down-regulated *TdDRF1* transcripts was also calculated using molecular data as a fixed factor and agronomic data (GY and TKW) as the variable ones.

## 3. Results

### 3.1. Agronomic Data

The grain yield (GY) and thousand kernel weight (TKW) averaged over four replicates in the two irrigation conditions are reported in Table 3.

### 3.2. Expression Profiles of TdDRF1 Gene

For each transcript, ANOVA was carried out using a log transformation of normalized relative quantities (NRQs) of transcript between the two irrigation conditions (FI and RI) for each time. Significant differences are summarized in Table 4. With regard to the putative transcription factors, significant differences were found in the *TdDRF1.3* transcript at T1, in both *TdDRF1.1* and *TdDRF1.3* transcripts at T4, in the *TdDRF1.1* transcript at T5, and in the *TdDRF1.3* transcript at T7.

**Figure 1:**
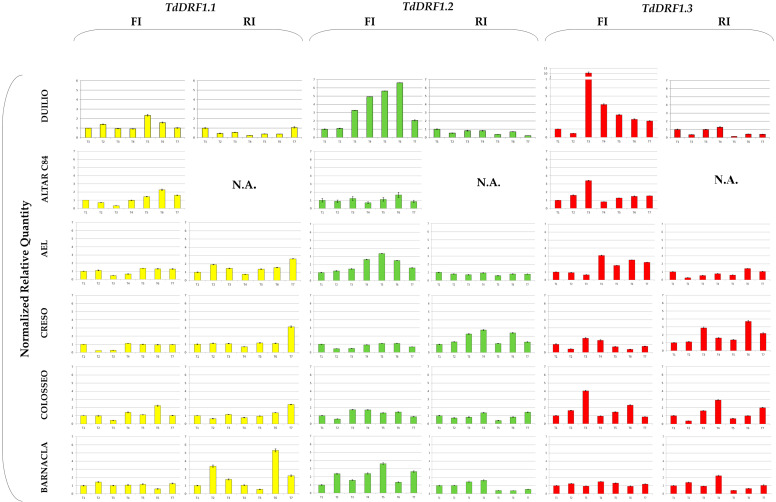
Expression profiles of the *TdDRF1* gene transcripts. Each expression profile is shown as normalized relative quantity rescaled to its own T1 value. Results referred to the six durum wheat genotypes analysed under both FI and RI conditions (*TdDRF1.1* in yellow, *TdDRF1.2* in green and *TdDRF1.3* in red, respectively). The time course consisted of seven points (T1 to T7). N.A., data not available. Data are represented as mean + SE.

For each genotype, NRQs of each transcript calculated during the time-course and rescaled to its own T1 value are shown in Figure 1. Unfortunately, the samples of Altar C84 under the RI condition were lost. As each profile value referred to its own T1, the analyses of transcripts were carried out at a trend level throughout the time-course. Furthermore, the strategy of relative quantification is only suitable for comparing results from the same transcript between treatments, so that results obtained with primer pairs different to each other could not be directly compared [20].

Comparing the genotypes shown in Figure 1, the *TdDRF1.1* transcript under the FI condition showed a slightly variable trend with some exceptions: Altar C84, AEL, Creso, and Colosseo showed a decrease at T3, while Duilio, Altar C84, AEL, and Colosseo showed an increase at T6. On the other hand, under the RI condition, each genotype showed a more distinct behaviour: Duilio showed a clear decrease at each time, AEL, Creso, and Colosseo remained almost constant at their T1 values, with a substantial increase at T7, while Barnacla showed a higher variability during the time-course with two large increases at T2 and T6.

With regard to the *TdDRF1.2* transcript under the FI condition, there were differences mainly at T3, T4, and T5. Duilio, AEL, and Barnacla displayed a large increase in comparison with their T1 values, while Altar C84 and Creso remained almost constant and Colosseo showed a slight increase. Under the RI condition, only Creso showed appreciable variations during the time-course.

As regards the *TdDRF1.3* transcript under the FI condition, all genotypes were found to be appreciably variable during the time-course with the exception of Barnacla. In particular, at T3 there was a large increase in Duilio, Altar C84, and Colosseo and, to a lesser extent, Creso, while AEL was late, showing an increase at T4. Under the RI condition, Duilio and AEL were almost constant or less than their initial values, while the other three genotypes showed variations, mainly in Creso and Colosseo and, to a minor extent, in Barnacla.

In addition, a tentative comparison of the trends among the three transcripts was carried out for each genotype. Duilio showed an overall expression of the three transcripts under full irrigation, while under reduced irrigation it seemed to be down-regulated by the water stress. Under full irrigation, Altar C84 showed a complementary activation of the transcription factors (*TdDRF1.1* and *TdDRF1.3*) at different times. AEL showed similar behaviours for the *TdDRF1.2* and *TdDRF1.3* transcripts under full irrigation, while under reduced irrigation neither transcript showed any appreciable changes, with *TdDRF1.1* being variable. Creso showed an almost constant expression of the three transcripts under full irrigation, while under reduced irrigation the *TdDRF1.2* and *TdDRF1.3* transcripts were found to respond to the water stress. On the other hand, Colosseo expressed mainly the *TdDRF1.3* transcript under both conditions, while Barnacla expressed only the *TdDRF1.2* transcript under full irrigation and the *TdDRF1.1* transcript under reduced irrigation with a single peak in the *TdDRF1.3* transcript at T4.

**Figure 2:**
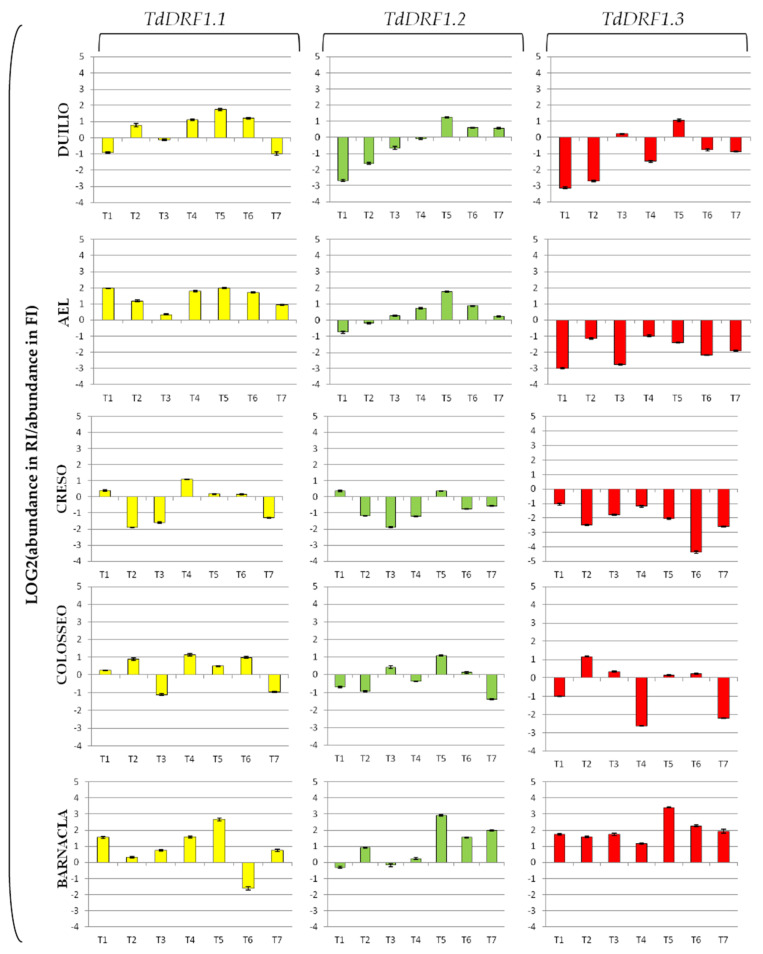
Expression profiles of the three *TdDRF1* transcripts. Each expression profile is shown as fold change (log2 value of the ratio for abundance under stress and abundance under control). Results referred to five durum wheat genotypes analysed under both FI and RI conditions (*TdDRF1.1* in yellow, *TdDRF1.2* in green, and *TdDRF1.3* in red, respectively). The time course consisted of seven points (T1 to T7). Data are represented as mean ± SE.

The expression profiles represented as fold change (log2 value of the ratio for abundance under stress and abundance under control) for each transcript are shown in Figure 2. The *TdDRF1.1* transcript turned out to be mostly up-regulated in the genotypes, particularly at T4, T5, and T6, to different extents, with Creso showing the lowest values. The *TdDRF1.2* transcript showed the greatest variability among genotypes, even if all of them were up-regulated at T5. Both AEL and Duilio showed a similar trend, initially down-regulated and then shifting to up-regulated at different time points (T3 and T5, respectively). Creso was mostly down-regulated, while Colosseo showed a swinging trend (up- and down-regulated at different times). Barnacla was largely up-regulated from T5 onwards. The *TdDRF1.3* transcript was largely up-regulated in Barnacla during the whole time-course with a peak at T5 (in fact detectable in all its transcripts). On the contrary, all other genotypes showed a down-regulation throughout the time-course, apart from a light up-regulation in Colosseo and Duilio at T2 and T5, respectively.

### 3.3. Statistical Association between Fold Change of TdDRF1 Transcripts and Traits

With the aim of finding an association between the agronomical data and the fold change in the three transcripts under the reduced irrigation condition, molecular data were represented using a binary code. For each genotype, we assigned a value = 0 representing a down-regulation and a value = 1 representing an up-regulation at each time point, whatever the size (see Figure 2). The analysis was carried out at the most relevant times for each trait analysed, that is, T2 for grain yield (GY) and T6 for thousand kernel weight (TKW), and was extended to neighbour points T1 and T7, respectively. The contrast between the molecular data (up- and down-regulation of transcripts) and the traits analysed is shown in Table 5.

At T2, the *TdDRF1.1* transcript was significantly associated to GY, with *p* < 0.01 and all up-regulated genotypes showing a mean GY significantly higher than Creso (down-regulated). *TdDRF1.2* was also significantly associated (*p* < 0.01) to the yield and Barnacla (up-regulated) showed a GY value significantly higher than the mean GY of all others. On widening the analysis to T1, Barnacla, characterized by up-regulation of the *TdDRF1.3* transcript, again showed a GY value significantly higher than all the others.

Considering the other agronomic trait, TKW, a significant association was found at T6 for *TdDRF1.2*, and the down-regulated genotypes (Creso and Barnacla) showed a TKW value significantly higher than that of the up-regulated group (Duilio, AEL, and Colosseo). On the other hand, widening the analysis to T7, *TdDRF1.1* was also significantly associated to TKW and the group of down-regulated genotypes (Duilio, Creso, and Colosseo) showed a mean TKW value significantly higher than the up-regulated group (AEL and Barnacla).

### 3.4. Expression Profile of the Wdhn13 Gene

**Figure 3:**
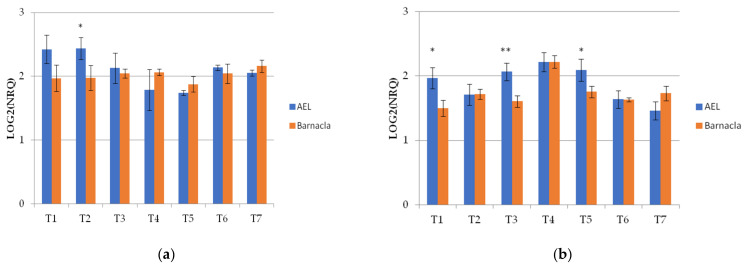
The expression profiles of *Wdhn13* gene transcript in the two genotypes measured under FI and RI conditions. (**a**) The expression profiles of *Wdhn13* transcript in AEL and Barnacla observed under FI condition. (**b**) The expression profiles of *Wdhn13* transcript in AEL and Barnacla observed under RI condition. Data are represented as mean ± SE. Significance codes: ** *p* < 0.01, * *p* < 0.05.

The *Wdhn13* gene encodes for LEA D-11 DHN (dehydrin), which has been reported to respond to water stress in wheat plants [21,24]. The *Wdhn13* gene transcript was analysed in two genotypes, AEL and Barnacla, under both irrigation conditions. Regardless of genotypes, significant differences of the *Wdhn13* gene transcript were found between FI and RI conditions at T1, T2, T4, T6, and T7, as shown in Table 6. The expression profiles of the *Wdhn13* gene transcript obtained analysing the environments separately in two genotypes are reported in Figure 3. Figure 3a refers to the full irrigation condition, while Figure 3b refers to the reduced irrigation condition. No significant differences were found between genotypes under FI, with the exception of T2 (*p* < 0.05), while significant differences were observed under RI (at T1, T3, and T5).

**Figure 4:**
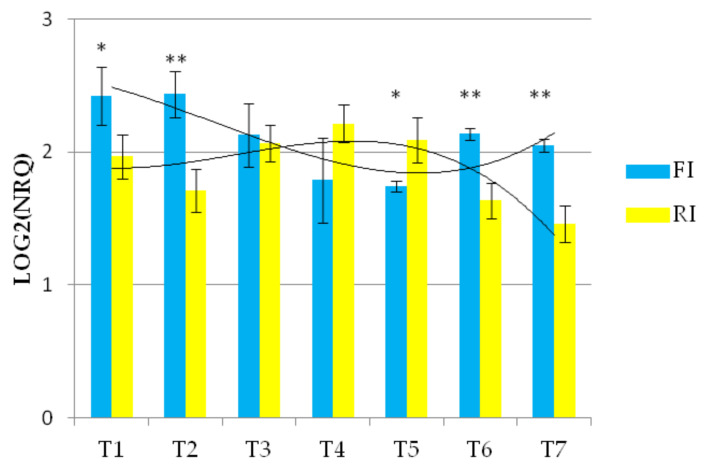
The expression profile of *Wdhn13* gene transcript of AEL under FI and RI conditions. A third-order polynomial trendline was added to highlight the opposite concavities; ** *p* < 0.01, * *p* < 0.05.

The expression of the *Wdhn13* gene transcript in AEL is shown in Figure 4 and demonstrated significant differences under the two conditions, showing almost opposite concavities crossing at T3 and T6, suggesting up-regulation under reduced irrigation.

**Figure 5:**
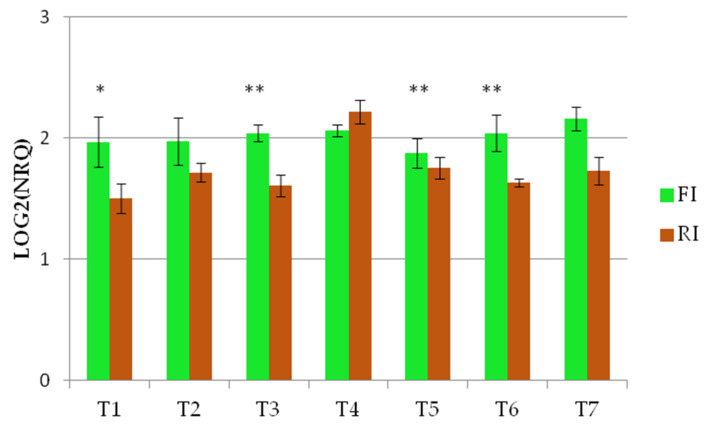
The expression profile of *Wdhn13* transcript of Barnacla under FI and RI conditions ** *p* < 0.01, * *p* < 0.05.

The *Wdhn13* gene transcript profiles were also different between the two conditions in Barnacla, showing a larger variability under reduced irrigation, while the expression levels under full irrigation were almost stable (Figure 5).

## 4. Discussion

This study was aimed at analysing and comparing the expression levels of the *TdDRF1* gene in the field under two different irrigation conditions, i.e., full and reduced irrigation (FI and RI, respectively), taking into account the reported involvement of its transcripts under water stress. Six different durum wheat genotypes were studied with the aim of finding any different behaviours among the transcripts. Our results revealed significant differences in the *TdDRF1* transcripts between the two irrigation conditions at different time points (Table 4). Both the *TdDRF1.1* and *TdDRF1.3* transcripts showed significant differences between the two conditions at T4, suggesting their full involvement in drought response, which continued with *TdDRF1.1* at T5 and *TdDRF1.3* at T7, pointing to their possible different role in the *trans*-activation throughout the time. On the other hand, these results could reflect the activity of the alternative splicing mechanism during the time-course, producing and cumulating the three transcripts in different ways as a response to the environment [25]. The modulation of the *TdDRF1.2* transcript is particularly interesting, as the latter is not directly involved in the downstream gene modulation but, with high probability, plays an important role in gene regulation through its expression and degradation via nonsense-mediated mRNA decay or other RNA surveillance mechanisms during transcript maturation, as reported for the abortive forms produced by the alternative splicing mechanism [26,27].

Looking at the trends shown in Figure 1 as a whole, every genotype seemed to have its own transcripts profile, depending mainly on genetics rather than environmental effects, as was previously observed [11,12,28]. The *TdDRF1.1* transcript remained almost steady across the time-course in both conditions, with few exceptions other than the notable behaviour of Barnacla under the reduced irrigation condition. Since the *TdDRF1.1* transcript results from the junction of all four exons (E1-E2-E3-E4) present in the gene sequence, it could be speculated that the quantity of this transcript, as the first product obtained, remains lower than the other ones, taking into account another mechanism very common in plants, known as intron retention [29], which could explain the formation of *TdDRF1.2* (E1-E2-E4) and *TdDRF1.3* (E1-E4) transcripts from an immature *TdDRF1.1* transcript. In addition to a relatively high value for the *TdDRF1.1* transcript, Barnacla also showed an opposite behaviour to the others under reduced irrigation. In the other genotypes, the *TdDRF1.3* transcript under full irrigation increased throughout the time-course, with some exceptions and to different extents, with Duilio up to 10-fold. Under reduced irrigation, Duilio and AEL did not change their initial expression levels, while both Creso and Colosseo showed substantial increases. It is important to underline that all the results are based on a time-course spanning 124 days from sowing with a seven-day window (Table 2); it could be possible that, in some cases and particularly under reduced irrigation, the window was too large to capture the expression modulations based on early and short responses. The *a priori* selection of an experimental design aimed at analysing gene expression in the field is a challenging task, particularly when the study involves transcription factors whose few molecules are sufficient to respond promptly to the stress by producing a large transcriptional burst of stress-responsive gene expression in a short period. Furthermore, in the field, plants may experience several distinct abiotic stresses either concurrently or at different times through the growing season [30], and consequently plant responses to water scarcity are very complex [31,32]. Finally, durum wheat is an allopolyploid (tetraploid) species and, due to its large genome size and the high levels of sequence similarity between the chromosomes duplicated, it is traditionally difficult to analyse [33]. Undeniably, for all the above-mentioned reasons, the number of gene expression studies using plant materials from the field is much less covered by the scientific literature than those under controlled environmental conditions.

With the results represented as the fold change, it is possible to state the expression levels in terms of down- and up-regulation of transcripts. It is worth noting that Duilio and AEL showed similar trends for all transcripts with very few differences: *TdDRF1.1* was always up-regulated, while *TdDRF1.3* was always down-regulated at all times. This molecular behaviour may suggest that the early stress response, aimed at activating *in trans* downstream genes, is mainly based on the 1.1 isoform. On the whole, the three transcripts of *TdDRF1* in Creso were strongly inhibited by water stress, possibly suggesting that this genotype employs other genes to cope with drought stress. Colosseo showed a certain similarity with Duilio and AEL with regard to the *TdDRF1.1* transcript. The molecular behaviour of the *TdDRF1* gene in Barnacla was unique, as all transcripts were largely up-regulated, suggesting a continuous transcription process that cumulates the three transcripts through a very articulated and complex control of alternative splicing [34]. Interestingly, in the contrast between the molecular and agronomic data, the up-regulation of the *TdDRF1.2* and *TdDRF1.3* transcripts, at T2 and T1, respectively, was significantly associated with grain yield, with Barnacla showing a value higher than the mean of all the others. The significant associations found by the statistical analyses are intriguing, but further studies at a molecular level and with a larger panel of different genotypes are necessary to better clarify the role of the transcripts in field stress response and to monitor their possible effects on the different stages of plant development and maturation.

A further aim of this work was to highlight the possible relationship between the *TdDRF1* transcripts and the *Wdhn13* gene, involved in environmental stress tolerance. In a recent study, Mehrabad Pour-Benab and colleagues [35] investigated dehydrin expression in different species of *Triticum* and *Aegilops* under well-watered and drought stress treatments in the greenhouse. Thirty days after applying the water stress, they observed the doubling of the *Wdhn13* expression level in water-stressed plants in the different wheat species. Our analysis in the field showed significant differences in the *Wdhn13* transcript levels between FI and RI conditions, at every time point except T3 and T5. Similarly, the *TdDRF1* transcripts were also found to be significantly different between the two conditions throughout the time-course. It could be speculated that the largest *Wdhn13* differences at T6 and T7 reflect the activity of transcription factors (*TdDRF1.1* at T5 and *TdDRF1.3* at T7) on the promoter of the *Wdhn13* gene, resulting finally in the dehydrin protein, involved in the drought stress tolerance at a cellular level.

Our results showed that the molecular behaviour of specific plant genes is highly dynamic and much more complex than a direct on/off switching. Furthermore, they confirm the main effect of genotype on the *TdDRF1* gene expression profile and, in this regard, the molecular behaviour of Barnacla appears particularly interesting, as it is the only genotype showing both transcription factors active under reduced irrigation during the time-course, even if to different extents. It is particularly intriguing that the grain yield value of Barnacla was the highest and was statistically associated with the up-regulation of two transcripts under reduced irrigation, thus conferring an added value to this genotype.

In conclusion, notwithstanding the intrinsic difficulty of identifying the effect of a single factor in the complex picture of gene expression, this work represents a remarkable contribution to highlighting the role of the *TdDRF1* gene in field conditions. Further experiments are necessary with a larger panel of genotypes and set of genes (transcription factors and target genes) to better understand the molecular plant response to drought, with the final aim of setting up a support tool to assist in orienting breeders’ decisions.

## Figures and Tables

**Table 1:** Pedigree information, date of release/development, and origin of six durum wheat genotypes (Country of origin: IT, Italy; MX, Mexico).

Genotype Name	Pedigree	Year of Release or Development	Country of Origin
Duilio	Cappelli//Anhinga/Flamingo	1984	IT
Altar C84	Ruff/Free Gallipoli/2/Mexicali75/3/Shwa	1985	MX
AEL *	Gediz//Fgo//Gta/3/Srn_1/4/Totus/5/Ente/Mexi_2//Hui/3/Yav_1/Gediz/6/Sombra_20/7/Stot//Altar 84/Ald	2005 **	MX
Creso	CpB144×[(Yt54N10B)Cp263Tc3]	1974	IT
Colosseo	Mexa/Creso Mutant	1994	IT
Barnacla	Arment//Srn_3/Nigris_4/3/Canelo_9.1	2003 **	MX

* AEL: Abbreviation used by authors for this advanced experimental line; ** year of development of the breeding line, not yet registered or commercially released.

**Table 2:** Leaf collection schedule implemented during the time-course experiment to establish the expression profile of the *TdDRF1* gene.

Time-Course	Weeks after T1	Month
T1 (1st collection date)	82 days after sowing	End of January
T2 (2nd collection date)	1	Early February
T3 (3rd collection date)	2	February
T4 (4th collection date)	3	February
T5 (5th collection date)	4	End of February
T6 (6th collection date)	5	Early March
T7 (7th collection date)	6	March

**Table 3:** Grain yield (GY) in *ton/ha* and thousand kernel weight (TKW) in *g* observed for the six durum wheat genotypes, evaluated under full (FI) and reduced (RI) irrigation conditions. Data reported are means ± standard deviations.; * *p* < 0.05; ** *p* < 0.01.

Genotype	FI		RI	
	GY *	TKW	GY **	TKW **
Duilio	6.6 ± 0. 44 (ab)	48.2 ± 2.08 (a)	2.5 ± 0.45 (bc)	41.0 ± 1.0 (bc)
Altar C84	6.6 ± 0. 85 (ab)	41.8 ± 2.89 (a)	1.4 ± 0.08 (a)	43.5 ± 0.50 (c)
AEL	7.1 ± 0. 86 (b)	41.8 ± 1.89 (a)	2.4 ± 0.23 (bc)	34.3 ± 1.44 (a)
Creso	5.3 ± 0. 61 (a)	44.0 ± 4.09 (a)	1.8 ± 0.23 (ab)	46.7 ± 1.53 (d)
Colosseo	5.9 ± 0.01 (ab)	48.8 ± 2.02 (a)	2.4 ± 0.36 (bc)	40.2 ± 1.26 (b)
Barnacla	5.8 ± 0. 68 (ab)	41.8 ± 1.26 (a)	3.1 ± 0.62 (c)	40.0 ± 0.50 (b)

Means with the same letter are not significantly different.

**Table 4:** ANOVA summery table. Significant differences in *TdDRF1* transcripts between FI and RI conditions during the time-course are shown.

Time	Transcript	F	*p*
T1	*TdDRF1.2*	F(1,29) = 8.35	<0.01
	*TdDRF1.3*	F(1,17.47) = 10.78	<0.01
T2	*TdDRF1.2*	F(1,20.88) = 10.5	<0.01
T4	*TdDRF1.1*	F(1,31) = 9.86	<0.01
	*TdDRF1.3*	F(1,31) = 10.33	<0.01
T5	*TdDRF1.1*	F(1,31) = 4.22	<0.05
	*TdDRF1.2*	F(1,29) = 11.06	<0.01
T7	*TdDRF1.3*	F(1,31) = 18.75	<0.001

F, sample F statistics; *p*, significance.

**Table 5:** Statistical association between molecular and agronomical data under RI condition. Molecular data were represented as a binary code and agronomical traits are referred to as GY and TKW.

Trait	Time	Transcript	Estimated LSM				
			Genotypes	0 Group	Genotypes	1 Group	*p* = |*t*|
GY	T1	*TdDRF1.2*	All others	2.62	Creso	1.79	<0.05
	T1	*TdDRF* *1.3*	All others	2.28	Barnacla	3.14	<0.01
	T2	*TdDRF* *1.1*	Creso	1.79	all others	2.62	<0.01
	T2	*TdDRF1.2*	All others	2.28	Barnacla	3.14	<0.01
TKW	T6	*TdDRF* *1.2*	Creso-Barnacla	43.33	Duilio-AEL- Colosseo	38.50	<0.01
	T7	*TdDRF* *1.1*	Duilio-Creso- Colosseo	42.61	AEL-Barnacla	37.17	<0.01
	T7	*TdDRF* *1.2*	Creso-Colosseo	43.42	Duilio-AEL- Barnacla	38.44	<0.05

LSM, Estimated least square means; *p*, significance.

**Table 6:** ANOVA summery table. Significant differences in *Wdhn13* gene transcript between FI and RI conditions during the time-course.

Time	F	*p*
T1	F(1,10) = 7.13	<0.05
T2	F(1,10) = 14.1	<0.01
T4	F(1,10) = 6.67	<0.05
T6	F(1,9) = 138.06	<0.0001
T7	F(1,10) = 36.86	<0.0001

F, sample F statistics; *p*, significance.

## Data Availability

Not applicable.

## Supplementary Materials

The following supporting information can be downloaded at: https://www.mdpi.com/article/10.3390/genes13030555/s1, Figure S1: Specific amplification and detection of the three *TdDRF1* transcripts, Figure S2: Schematic representation of the complete procedure of reverse transcription, pre-amplification, and qRT-PCR, Table S1: Gene-stability values (M) of the analysed reference genes.

## Abbreviations

AEL, Advanced experimental line; DRE, *drought-responsive element*; *DREB,* dehydration responsive element binding gene; FC, fold change; FI, full irrigation; GY, grain yield; NRQ, normalized relative quantity of transcript; RI, reduced irrigation; *Td**DRF1*, *Triticum durum* dehydration responsive factor 1 gene; TKW, thousand kernel weight; TF, transcription factor; *Wdhn13,* wheat dehydrin 13 gene.
