# Supplementary material for: Expression Analysis of the TdDRF1 Gene in Field-Grown Durum Wheat under Full and Reduced Irrigation
Source: Genes (Basel). 2022 Mar 21;13(3):555. doi: 10.3390/genes13030555 (PMC8953156; doi:10.3390/genes13030555)
Supplement: Supplementary file 1 [file genes-13-00555-s001.zip › genes-1624059-Supplementary.pdf]

Supplementary Materials

Supplementary Figure S1: Specific amplification and detection of the three *TdDRF1* transcripts.

On the left: experimental design of TaqMan primers and probes for the specific amplification and detection of the three *TdDRF1* gene transcripts. “R” is the “Reporter” dye attached at the 5′-end of the probe sequence and “Q” is the non-fluorescent “Quencher” on the 3′-end. E1, E2, E3, E4 indicate Exon 1, 2, 3, 4.
On the right: end-point RT-PCRs run on 3% agarose gel confirming the specificity of primers and probes (by the length and further band sequencing). The gel refers to Duilio RI samples collected at T3.

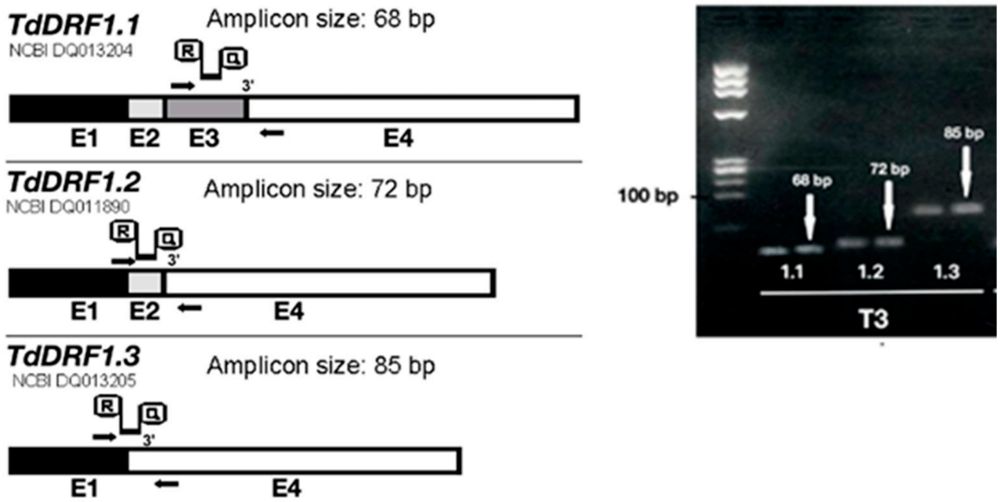

Supplementary Figure S2: Schematic representation of the complete procedure of reverse transcription, pre-amplification and qRT-PCR.

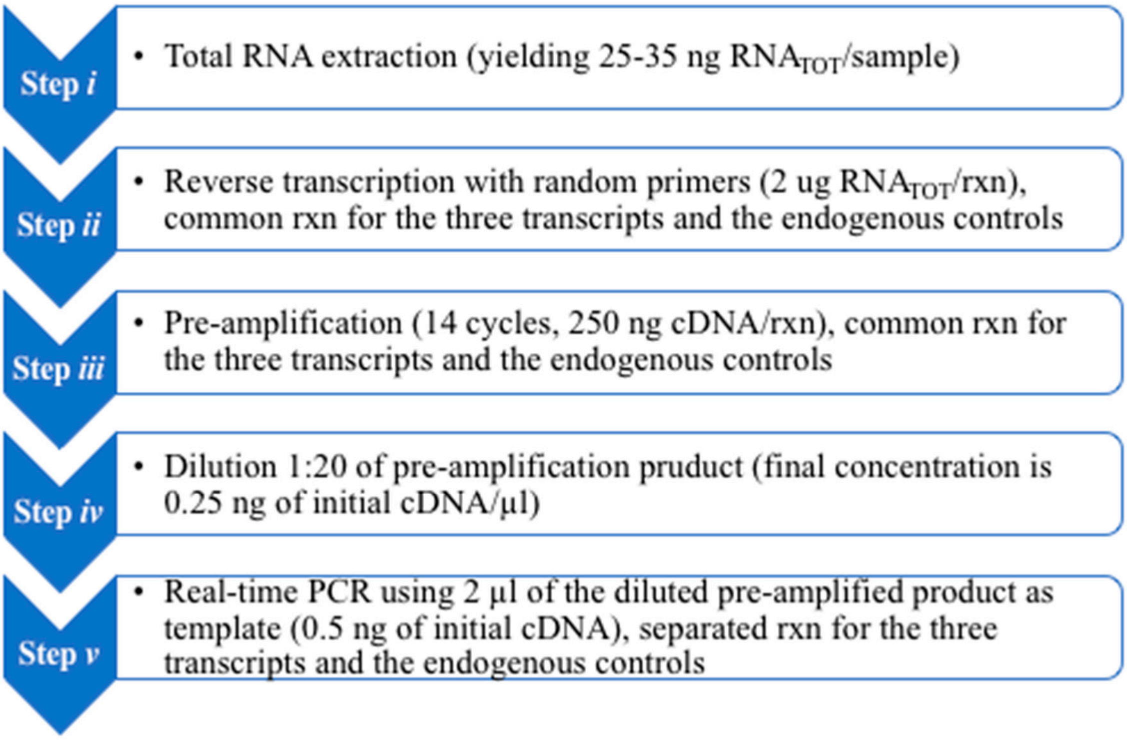

Supplementary Table S1: Gene-stability values (*M*) of the analysed reference genes

**Housekeeping gene**	***M***
18S rRNA	0.980
Actin	0.905
Ta2291	0.936
Ta2776	0.985
TaSnK1	0.802
